# MDMX phosphorylation-dependent p53 downregulation contributes to an immunosuppressive tumor microenvironment

**DOI:** 10.1093/jmcb/mjaa038

**Published:** 2020-07-24

**Authors:** Bing Wang, Chuan-Bian Lim, Jiawei Yan, Lizhen Li, Jufang Wang, John B Little, Zhi-Min Yuan

**Affiliations:** 1 Key Laboratory of Space Radiobiology of Gansu Province & Key Laboratory of Heavy Ion Radiation Biology and Medicine of Chinese Academy of Sciences, Institute of Modern Physics, Chinese Academy of Sciences, Lanzhou, China; 2 University of Chinese Academy of Sciences, Beijing, China; 3 John B. Little Center for Radiation Sciences, Harvard T.H. Chan School of Public Health, Boston, MA, USA

**Keywords:** p53, MDMX, tumor microenvironment, immune cell infiltration, macrophage polarization

## Abstract

A role of tumor-suppressive activity of p53 in the tumor microenvironment (TME) has been implicated but remains fairly understudied. To address this knowledge gap, we leveraged our *Mdmx^S314A^* mice as recipients to investigate how implanted tumor cells incapacitate host p53 creating a conducive TME for tumor progression. We found that tumor cell-associated stress induced p53 downregulation in peritumor cells via an MDMX-Ser314 phosphorylation-dependent manner. As a result, an immunosuppressive TME was developed, as reflected by diminished immune cell infiltration into tumors and compromised macrophage M1 polarization. Remarkably, ablation of MDMX-Ser314 phosphorylation attenuated p53 decline in peritumor cells, which was associated with mitigation of immunosuppression and significant tumor growth delay. Our data collectively uncover a novel role of p53 in regulating the tumor immune microenvironment, suggesting that p53 restoration in the TME can be exploited as a potential strategy of anticancer therapy.

## Introduction

Ample evidence indicates that the tumor microenvironment (TME) plays a crucial role in tumor development and progression (Wang et al., 2017). The TME is a complex network that is composed of cancer cells, stromal and immune cells, extracellular matrix, and secreted factors such as cytokines and chemokines (Quail and Joyce, 2013), which can profoundly modulate the functions of immune cells and critically contribute to immunosuppression (Vijayan et al., 2017). An improved understanding of how tumor cells can create an immunosuppressive TME would aid in the development of therapeutic strategies.

As one of the most important tumor suppressors, p53 is universally inactivated in human cancers (Kastenhuber and Lowe, 2017). Approximately 50% of all human cancers have mutations in the *TP53* gene, which occurs largely within the DNA-binding domain. Loss of DNA binding invariably abrogates p53’s ability to suppress tumorigenesis. Apart from the *TP53* gene mutations, p53 function can be compromised alternatively by a reduction in nuclear p53 levels. In line with this notion, oncogenic signaling-induced cell proliferation is accompanied with p53 downregulation (Lei et al., 2011; Zwang et al., 2011). A decline in p53 levels due to overexpression of the key p53 E3 ligase MDM2 or MDM2/MDMX is frequently found in many human cancers. In addition to overexpression/amplification, the level and/or activity of MDM2/MDMX can be enhanced by a mechanism of post-translational regulation (Wasylishen and Lozano, 2016). Given its localization, MDMX, rather than MDM2, is often the first recipient of oncogenic signaling cues directed toward the MDM2/MDMX complex and aimed at suppressing p53. Although ultimately both MDM proteins are required for an effective modulation of p53, they have been shown to display non-overlapping functions, which could also include their role in mediating upstream signaling pathways targeted at the p53 core. MDMX represents a ‘signaling hub’ transducing oncogenic signals as well as stress signals to the MDM2/MDMX complex and therefore to p53 (de Polo et al., 2016). In this context, oncogenic receptor tyrosine kinases can inhibit p53 activation via modulation of the MDM2/MDMX complex stability and, specifically, through post-translational modifications of MDMX (Gerarduzzi et al., 2016). The mechanism of post-translational regulation in p53 suppression is more prevalent in the TME because in a sharp contrast with tumors, p53 mutation in stromal cells is fairly rare. Apart from the receptor tyrosine kinases, our prior study also identified p38 as another protein kinase that enhances MDM2/MDMX-mediated p53 inhibition via targeting MDMX for phosphorylation (de Polo et al., 2017). Given the critical role of both tyrosine kinases (Tan et al., 2018) and p38 in stress response, we investigated in the current study MDMX phosphorylation in modulation of p53 function in the TME.

## Results

### Generation of Mdmx^S314A^ knockin mice

To evaluate stress-induced MDMX phosphorylation in a biologically relevant setting, we created a knockin mouse model expressing *Mdmx^S314A^* (Supplementary Figure S1A and C). The correct genotypes were determined using quantitative polymerase chain reaction (PCR) method and confirmed by sequencing (Supplementary Figure S1B and D). Pups of all genotypes were born according to the Mendelian ratio (Supplementary Table S3) and animals did not exhibit discernable phenotype (Supplementary Figure S1E). The data collectively indicate that expression of *Mdmx^S314A^* caused little perturbation of homeostasis under physiological conditions. Of note, in *Mdmx^S314A^* mice, both *p53* and *Mdm2* are completely unaltered or expressed in the natural wild-type form, representing an excellent model for investigating how MDMX phosphorylation affects MDM2/MDMX-mediated p53 regulation.

### Mdmx^S314A^ mice exhibit enhanced tumor control

**Figure 1:**
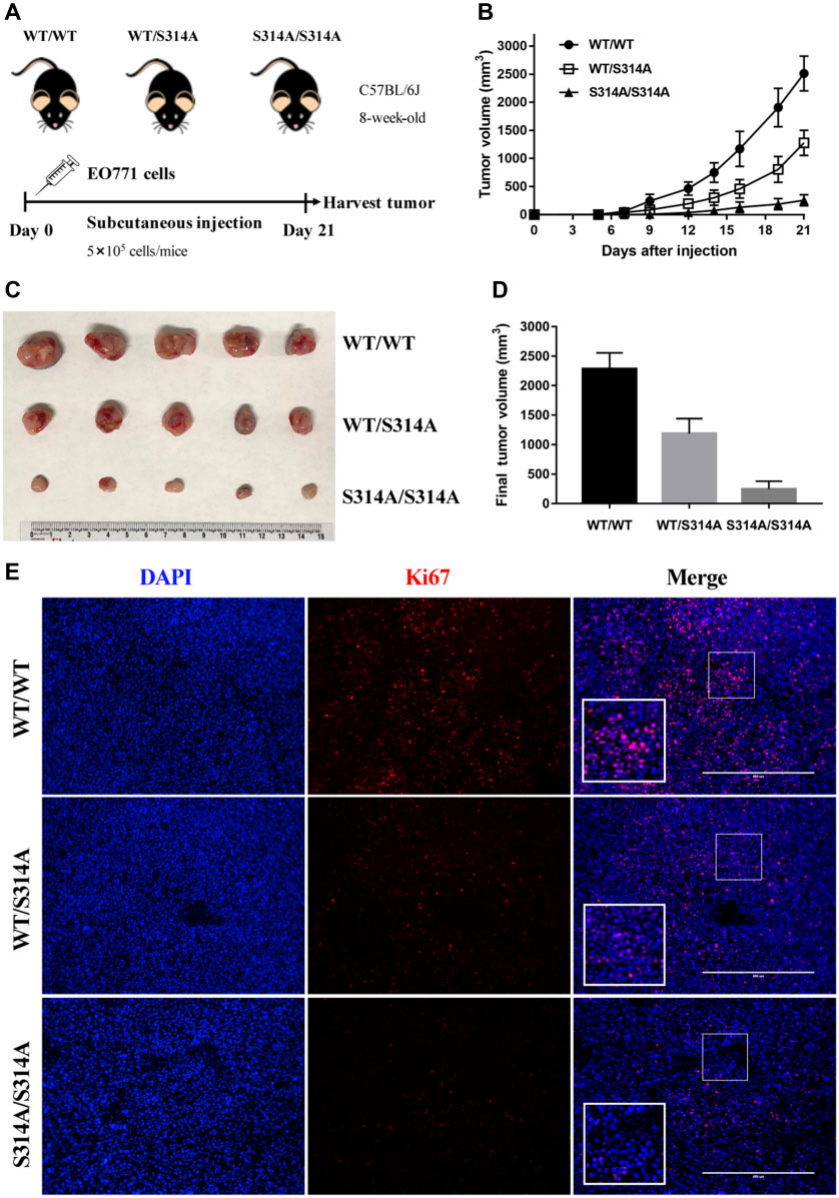
*Mdmx^S314A^* mice exhibit enhanced tumor control. (**A**) Experimental design scheme. EO771 cells were injected into the right flank of mice (n =* *5/genotype) on Day 0 and tumors were harvested on Day 21. Tumor size was measured by caliper every other day. (**B**) Growth curve of EO771 syngeneic tumors in different genotypic mice over 21 days. (**C**) Gross appearance of tumors derived from EO771 cells on Day 21 after implantation into wild-type, *Mdmx^WT/S314A^*, and *Mdmx^S314A/S314A^* mice. (**D**) Mean values of tumor volume in different genotypic mice on Day 21. (**E**) Immunofluorescence for Ki67 (red) and DAPI (blue) in EO771 tumor sections from wild-type, *Mdmx^WT/S314A^*, and *Mdmx^S314A/S314A^* mice. Scale bar, 400 μm.

The TME is typically hypoxic, acidic, and deficient in nutrients, which are associated with persistent stress (Bowser et al., 2017). As stress-responsive protein kinases, tyrosine kinases and p38 are frequently activated in the TME and have been implicated in promoting tumor progression (Hotamisligil and Davis, 2016; Olson et al., 2017; Suarez-Lopez et al., 2018; Tan et al., 2018). Having shown that both tyrosine kinases and p38 target MDMX for phosphorylation resulting in enhanced MDM2/MDMX-mediated p53 degradation (Gerarduzzi et al., 2016; de Polo et al., 2017), we sought to leverage the *Mdmx^S314A^* mice to functionally characterize stress-induced MDMX phosphorylation *in vivo*. In order to focus on the TME, we designed our study by employing a syngeneic mouse model of mammary carcinoma. In this context, murine EO771 medullary mammary adenocarcinoma cells, which are derived from C57BL/6J mice, were transplanted into *Mdmx^S314A^* mice and wild-type littermates (also from C57BL/6J background). As verified (Supplementary Figure S2), the EO771 cell line harbors a functional defective mutant p53 (ATCC), which is not only fairly aggressive but also unresponsive to regulation. Thus, MDMX phosphorylation-mediated p53 modulation would only be limited to the host microenvironment, enabling us to assess the interaction of host p53 with implanted EO771 cells. Equal number of proliferative EO771 cells (5 × 10^5^/mouse) was subcutaneously transplanted into the hind flank of wild-type, *Mdmx^WT/S314A^*, or *Mdmx^S314A/S314A^* mice. The implanted tumor growth was monitored for 21 days (Figure 1A). Remarkably, the growth of EO771 subcutaneous tumors was significantly delayed in *Mdmx^S314A^* mice when compared with the wild-type mice (Figure 1B). Of note was the gene dosage effect in that homozygous *Mdmx^S314A/S314A^* mice displayed a greater delay in tumor growth compared with heterozygous *Mdmx^WT/S314A^* mice. Direct measurement of isolated tumors confirmed a significant decrease of tumor size in *Mdmx^WT/S314A^* and *Mdmx^S314A/S314A^* mice (Figure 1C and D). In accordance with tumor growth, immunohistochemistry (IHC) of the tumor tissues detected an overt increase in cell proliferation marker Ki67 (Figure 1E). Among the three genotypes, the Ki67 signal in the tumor was highest in wild-type and lowest in *Mdmx^S314A/S314A^* with *Mdmx^WT/S314A^* in between, consistent with the gene dosage-dependent effect found in tumor control. The data collectively indicate that ablation of MDMX phosphorylation at Ser314 in recipient mice suppressed the tumor growth of implanted mammary adenocarcinoma cells.

### Mdmx^S314A^ mice exhibit resistance to p53 downregulation induced by implanted tumor cells

**Figure 2:**
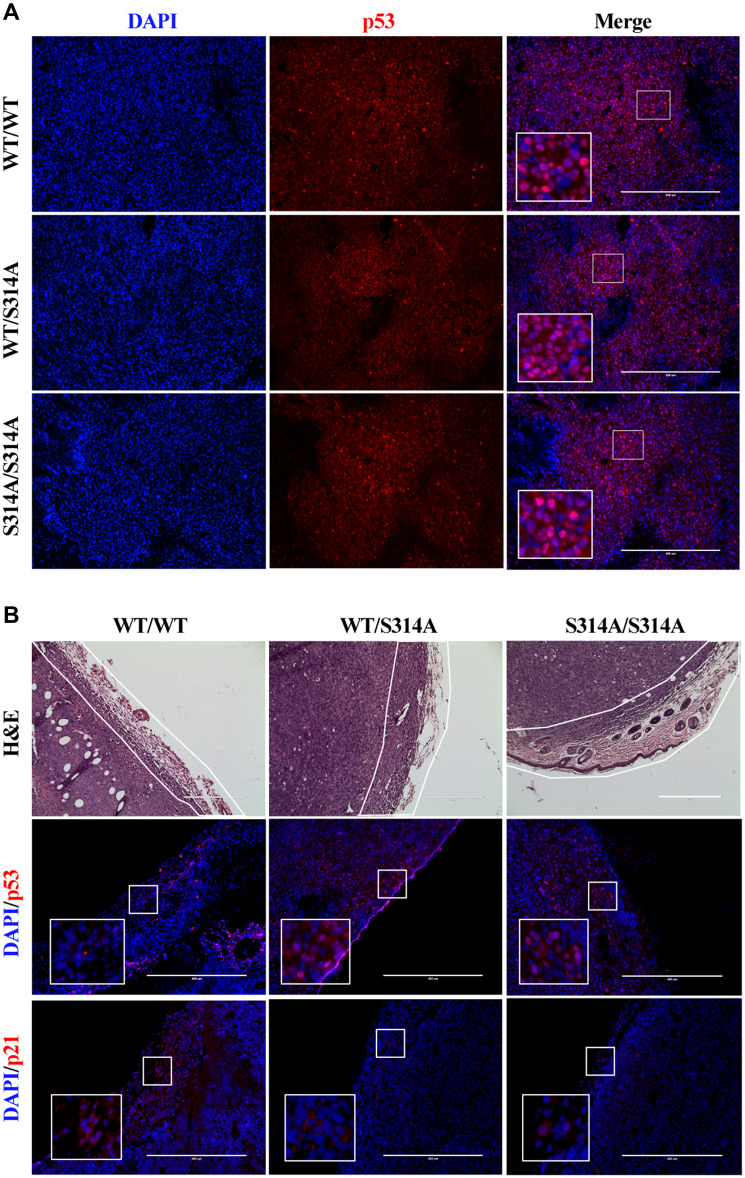
*Mdmx^S314A^* mice exhibit resistance to tumor cell-induced p53 downregulation. (**A**) Immunofluorescence for p53 (red) and DAPI (blue) in tumor sections from different genotypic mice. (**B**) Upper: hematoxylin and eosin (H&E)-stained images of tumor sections. The stromal tissues surrounding the tumor are outlined in white. Middle and lower: immunofluorescence for p53 (red, middle), p21(red, lower), and DAPI (blue) in stromal tissues from different genotypic mice. Scale bar, 400 μm.

We previously showed that phosphorylation of MDMX at Ser314 resulted in enhanced stability and activity of the MDM2/MDMX complex and subsequent p53 decline because of increased turnover (Gerarduzzi et al., 2016; de Polo et al., 2017). The resistance of *Mdmx^S314A^* mice to implanted tumor cells led us to examine a potential involvement of p53. There was a fairly strong p53 staining in tumor cells (Figure 2A), indicative of a high expression of the p53 mutant in EO771 cells. Little difference in the p53 signals within the tumors between mice of different genotypes was seen, confirming that the mutant p53 in implanted tumor cells was not affected by the host. However, in the surrounding stromal tissues, p53 was detectable, albert at a modest level, only in *Mdmx^S314A^* knockin but not wild-type mice (Figure 2B). IHC staining of p21 did not detect significant increase in *Mdmx^S314A^* mice (Figure 2B), indicating that while p53 levels were maintained, p53 was not transcriptionally activated. The data together revealed that abrogation of MDMX phosphorylation at Ser314 in the recipient mouse blocked implanted mammary adenocarcinoma cells-induced p53 downregulation in the TME.

### Mdmx^S314A^ mice display improved immune cell infiltration into tumors

**Figure 3:**
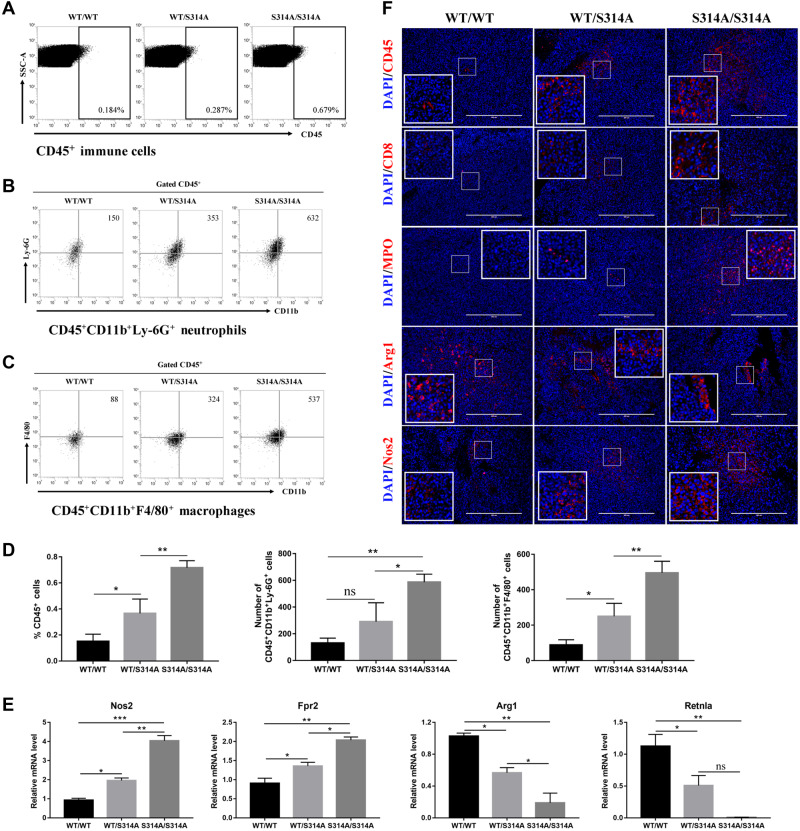
*Mdmx^S314A^* mice display improved immune cell infiltration and macrophage M1 polarization. (**A**‒**C**) Representative dot plots of immune cells (**A**), neutrophils (**B**), and macrophages (**C**) by flow cytometry in tumor tissues from wild-type, *Mdmx^WT/S314A^*, and *Mdmx^S314A/S314A^* mice. Immune cells are defined as CD45^+^ cells, neutrophils as CD45^+^CD11b^+^Ly-6G^+^ cells, and macrophages as CD45^+^CD11b^+^F4/80^+^ cells. (**D**) Quantitation of immune cells (left), neutrophils (center), and macrophages (right) by flow cytometry in tumor tissues from different genotypic mice. (**E**) Gene expression levels of *Nos2*, *Fpr2*, *Arg1*, and *Retnla* were determined by qPCR in tumor tissues from different genotypic mice (n=* *3/group). *Nos2* and *Fpr2* are M1 genes while *Arg1* and *Retnla* are M2 genes. (**F**) Immunofluorescence staining of tumor sections from different genotypic mice with indicated antibodies. Data shown are mean ± SEM; *P < 0.05, **P < 0.01, and ***P < 0.001 by one-way ANOVA followed by Tukey’s post hoc test. ns, non-significance. Scale bar, 400 μm.

Among many components in the TME, immune cells are known to play a central role in modulating tumor growth (Quail and Joyce, 2013; Wang et al., 2017). We thus asked whether the tumor growth delay observed in *Mdmx^S314A^* mice was mediated by the immune system. We addressed this question by examining tumor immune cell infiltration using fluorescence-activated cell sorting (FACS) to identify immune cell types in tumor homogenates. When compared among the three genotypes, there was a significant difference in the number of CD45^+^ immune cells in tumor tissues (Figure 3A and D). The gene dosage-dependent effect was very evident in that CD45^+^ cells were lowest in wild-type mice and the numbers increased in *Mdmx^S314A^* heterozygous mice and even further in homozygous mice (Figure 3D). Further characterization of CD45^+^ cells revealed that CD45^+^CD11b^+^Ly-6G^+^ neutrophils were one of the dominant populations. Again, neutrophil infiltration also showed a gene dosage effect similar to that seen with CD45^+^ cells (Figure 3B and D). The data together revealed a positive association between neutrophil infiltration and tumor growth delay.

Recent studies have shown that p38 MAPK promotes tumor progression by modulating the activity of tumor-associated macrophage (TAM) (Olson et al., 2017; Suarez-Lopez et al., 2018). We thus used antibodies specific to CD45^+^CD11b^+^F4/80^+^ to identify macrophages in tumor tissues. FACS analysis of CD45^+^CD11b^+^F4/80^+^ cells found that similar to neutrophils, macrophage infiltration into tumors was significantly higher in *Mdmx^S314A^* mice than that in wild-type littermates (Figure 3C and D). Macrophages can be broadly categorized into classical (M1) or alternative (M2), which have distinct roles in tumorigenesis (Quail and Joyce, 2017). We used M1- or M2-specific markers to analyze the infiltrated macrophages. The results indicated a dominant M1 polarization of the macrophages in *Mdmx^S314A^* mice as evidenced by a gene dosage-dependent increase in expression of M1 markers *Nos2* and *Fpr2*, which were notably paralleled by a significant reduction in expression of M2 markers *Arg1* and Resistin-like alpha (*Retnla*) (Figure 3E). The results indicate that inhibition of stress-induced MDMX phosphorylation was associated with stimulation of macrophage M1 polarization and concurrent blockage of M2 activation. To substantiate the data derived from FACS analyses, IHC was performed to detect immune cell infiltration into tumors by staining for immune cell type-specific markers: pan-immune cells (CD45), tumor-infiltrating lymphocytes (TIL, CD8), neutrophils (MPO), and M1 and M2 macrophages (Nos2 and Arg1, respectively). In agreement with the FACS data, the infiltration of immune cells including CD8^+^ T cells and neutrophils was evident, which were coupled with an increase in M1 marker Nos2 paralleled with a decrease in M2 marker Arg1 (Figure 3F). All these IHC markers exhibited changes in a gene dosage-dependent manner, corroborating the data from FACS analysis. Collectively, the results showed that the tumor growth delay in *Mdmx^S314A^* mice was associated with increased CD8^+^ T and neutrophils infiltration and macrophage M1 polarization when compared with that in wild-type mice.

### MDMX-Ser314 phosphorylation-mediated p53 modulation promotes macrophage M1 polarization

**Figure 4:**
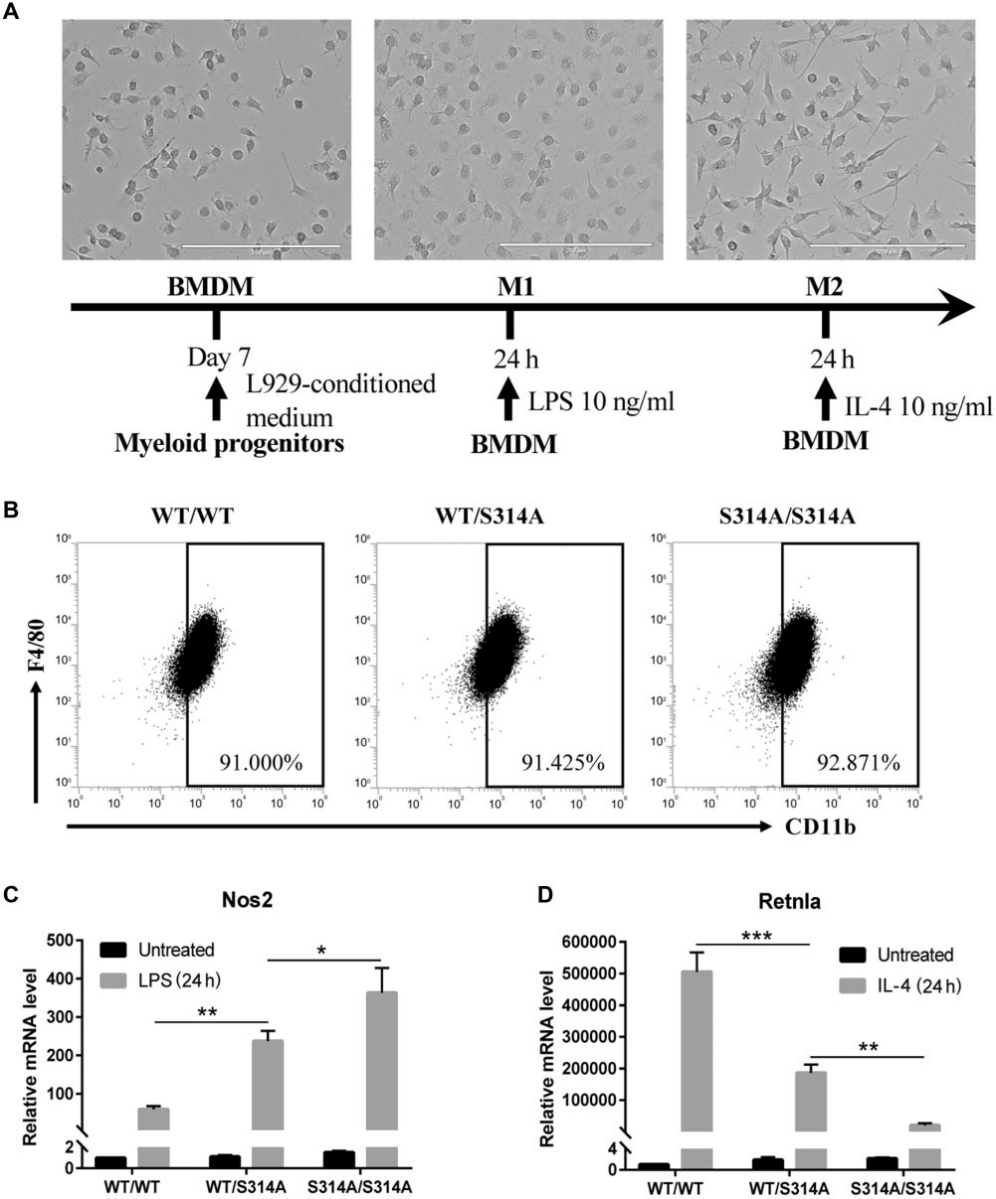
Mdmx-Ser314 phosphorylation-mediated p53 modulation promotes macrophage M1 polarization. (**A**) Schematic of method to induce BMDM polarization. Phase-contrast images of BMDMs untreated (left), treated with 10 ng/ml LPS for 24 h (center), and treated with 10 ng/ml IL-4 for 24 h (right) are shown. Scale bar, 200 μm. (**B**) FACS analysis of BMDMs from different genotypic mice. BMDMs were defined as CD11b^+^F4/80^+^cells. (**C** and **D**) Expression levels of M1 gene *Nos2* (**C**) and M2 gene *Retnla* (**D**) were determined by qPCR in BMDMs of different genotypes that were treated with LPS or IL-4 for 24 h. Data shown are mean ± SEM; *P < 0.05, **P < 0.01, and ***P < 0.001 by one-way ANOVA followed by Tukey’s post hoc test.

The above observations suggest that MDMX phosphorylation-dependent modulation of p53 is potentially involved in modulation of TAM. To further substantiate the observation, we isolated bone marrow-derived macrophages (BMDMs) from wild-type, *Mdmx^WT/S314A^*, and *Mdmx^S314A/S314A^* mice. Bone marrow-derived cells were differentiated into macrophages using a published standard protocol and the differential efficiency was measured (Figure 4A). The cells isolated from mice of the three genotypes exhibited comparable differentiation efficiency, with ∼90% CD11b^+^F4/80^+^ (Figure 4B), suggesting little direct effect of MDMX phosphorylation on macrophage differentiation.

We next assessed macrophage polarization. The M2 polarization was induced by IL-4 treatment and the expression of *Retnla*, which is a well-established murine M2 macrophage marker (Quail and Joyce, 2017), was significantly lower in *Mdmx^S314A^* macrophages than in wild-type controls (Figure 4D), suggesting a compromised M2 polarization when MDMX-Ser314 phosphorylation was blocked. We continued the analysis of macrophage polarization by treating bone marrow cells with an M1 inducer, bacterial lipopolysaccharide (LPS). The expression of *Nos2*, the hallmark of M1 macrophages, was monitored. The results revealed a marked enhancement of M1 polarization in bone marrow cells isolated from *Mdmx^S341A^* mice in a gene dosage-dependent manner (Figure 4C). The data are consistent with the *in vivo* results that blocking MDMX phosphorylation in *Mdmx^S314A^* mice was associated with M1 polarization.

## Discussion

Using syngeneic tumor xenograft mouse model, we show that upon implanting into a recipient mouse, tumor cells interact with their surroundings within the host resulting in marked p53 downregulation in peritumor cells. This p53 decline appears to be mediated by MDMX-Ser314 phosphorylation, since blocking this phosphorylation prevented p53 reduction. Of significance is the finding that maintaining p53 levels in the stromal cells, as shown in *Mdmx^S314A^* mice, resulted in marked tumor growth inhibition. The results collectively reveal an important tumor-suppressive function mediated by p53 in the peritumor cells.

Ample evidence indicates that cancer cell hyperproliferation is associated with not only cell-intrinsic stress such as genomic instability, oxidative stress, endoplasmic reticulum stress, and proteome stress but also profound perturbations in the surrounding, creating a TME that is usually featured with hypoxic, acidic, deficient in nutrients and inflammation (Quail and Joyce, 2013; Bowser et al., 2017). In response to the stress signals within the TME, p53 in the stromal cells may be stimulated or suppressed, dependent on not only the intensity but also duration of the stress signals. Of note is that we observed p53 downregulation rather than p53 activation in the peritumor cells, which suggests two possible scenarios. (i) The stress signals in the TME were relative mild and thus the intensity did not reach the threshold able to activate p53. In line with this notion, cell proliferation-induced stress is associated with p53 downregulation (Lei et al., 2011; Zwang et al., 2011). (ii) There is a range of stress signals with different intensities. Strong stress signals can activate p53 causing cell growth inhibition or death. These cells would be selected out during the course of TME development. Mild stress signals on the other hand would induce p53 reduction in the peritumor cells, rendering them survival advantage. As a result, the persistent stress in the TME can lead to accumulation of cells with diminished p53 levels. In agreement with our finding, a recent report demonstrated a correlation of MEK activation with compromised p53 activity in tumor-associated macrophages (Olson et al., 2017).

The *Mdmx^S314A^* mice enabled us to demonstrate that the decrease of p53 in the peritumor cells was mediated by, at least in part, by tumor-induced MDMX-Ser314 phosphorylation. Our previous work showed that MDMX-Ser314 was phosphorylated by CDK4/6 downstream of a number of tyrosine kinases and this site was also targeted by JNK/p38 (Gerarduzzi et al., 2016; de Polo et al., 2017). With abundant evidence indicating the contribution of these protein kinases to the tumorigenic effects in the TME (Hotamisligil and Davis, 2016; Olson et al., 2017; Suarez-Lopez et al., 2018; Tan et al., 2018), it is not unreasonable to speculate that p38 and tyrosine kinases might be responsible for the phosphorylation of MDMX-Ser314. Nonetheless, it does not exclude a potential involvement of other kinases since the TME is a very complex network that encompasses a range of dysregulated pathways. Further study will be necessary to fully characterize the TME for determining the pathways responsible for MDMX phosphorylation and p53 inhibition.

In light of the remarkable advance in our understanding of immunity-mediated tumor control, we explored how the reduction of p53 in the TME could affect the immune system. We observed that the decline of p53 in peritumor cells was coupled with significantly reduced tumor-infiltrating neutrophils and T cells, suggesting that p53 downregulation could contribute to the development of an immunosuppressive TME. Consistent with our finding, Guo et al. (2013) showed that when compared with the recipient mice harboring wild-type p53, implanted B16 melanoma cells grew much faster in p53-null host mice because of an immunosuppressive TME caused by p53 deficiency. Along a similar line, Walton et al. (2016) recently reported that p53 loss can induce increased expression of CCL2 that recruits immunosuppressive myeloid cells into the TME. Our work extended these findings by demonstrating that p53 in the peritumor cells was downregulated via a mechanism mediated by tumor-induced MDMX phosphorylation. Remarkably, impeding this p53 decline in the peritumor cells can mitigate the immunosuppressive TME, as indicated by the significant number of TIL and reduced tumor burden in *Mdmx^S314A^* mice.

We also assessed the innate immune components of the TME by examining tumor-infiltrating macrophages. Maintaining p53 levels in the TME, as shown in *Mdmx^S314A^* mice, was associated with not only marked increase of macrophage infiltration into tumors but also significant M1 polarization. The significant tumor growth delay observed in *Mdmx^S314A^* mice suggests a tumor-suppressive activity of M1 macrophages. Indeed, it has been reported that M1 macrophages contribute to the antitumor activity or elimination of cancer cells (Suarez-Lopez et al., 2018).

In conclusion, we showed that implanted tumor cells disturb their surroundings creating an immunosuppressive microenvironment that promotes tumor progression. This effect of tumor cells appears to be mediated by MDMX phosphorylation-dependent p53 downregulation in the host because blocking this MDMX phosphorylation prevented p53 decline and effectively mitigated the immunosuppressive TME, resulting in tumor growth delay. The work supports a tumor suppressor function of p53 in the TME, implicating that p53 restoration in the stromal cells may be a potential strategy of cancer intervention. Further studies are necessary to investigate how p53 can alleviate the immunosuppressive TME.

## Materials and methods

### Animals

All mouse strains were housed under specific pathogen-free conditions on a 12:12 light:dark cycle, with ad libitum UV-sterilized water and irradiated PicoLab Mouse Diet 20 (LabDiet).

### Generation of Mdmx^S314A^ mice

*Mdmx^S314A^* phosphomutant mouse was generated by Applied StemCell, Inc. on a C57BL/6J genetic background by replacing Ser314 with a non-phosphorylatable alanine by CRISPR–Cas9. We generated *Mdmx^WT/S314A^* heterozygous mice by crossing C57BL/6J mice with *Mdmx^S314A/S314A^* homozygous mice.

### Mouse genotyping

Genomic DNA was isolated from ear punch biopsies following 1‒16 h digestion at 55°C in lysis buffer containing 10 mM EDTA, 100 mM NaCl, 0.1% sodium dodecyl sulfate (SDS), 50 mM Tris-HCl (pH 8.0), and 0.1 mg/ml proteinase K (Thermo Scientific). PCR was performed using the following primers: 5ʹ-CTATGAAATTTGTTCAGGTCTCAGGTTGGAC-3ʹ (1F) and 5ʹ-CTCCTACAATCGGGAACATCAATTCCTTC-3ʹ (2R). The cycling protocol consisted of an initial denaturation at 95°C for 2 min followed by 35 cycles of 95°C for 30 sec, 60°C for 30 sec, and 72°C for 30 sec and a final extension at 72°C for 5 min. The resulting 360-bp amplicon was subsequently digested with the TaqI restriction enzyme (New England Biolabs), which cuts the phosphomutant allele resulting in two fragments (212 and 148 bp), and resolved on 2% agarose gel.

### Syngeneic mouse tumor model

EO771 cells were purchased from CH3 Biosystems LLC. Cells were grown in RPMI 1640 medium (Corning) supplemented with 10 mM HEPES (Gibco), 10% fetal bovine serum, 100 U/ml penicillin G, and 100 μg/ml streptomycin (Corning) and maintained at 37°C in a humidified 5% CO_2_ incubator. To establish the syngeneic breast cancer model, 5 × 10^5^ EO771 cells as 100 μl cell suspension in Dulbecco's phosphate-buffered saline (DPBS; Gibco) were injected into the hind flank of 8-week-old female mice. Caliper measurements of tumor size were taken every other day and used to calculate the tumor volume (V = L × W^2^/2).

### BMDM isolation and culture

BMDMs were isolated from 7- to 8-week-old mice and cultured as described previously to obtain naïve M0 macrophages (Dai et al., 2017). For M1 polarization, BMDMs were stimulated with 10 ng/ml LPS (Sigma Aldrich) for 24 h. For M2 polarization, BMDMs were stimulated with 10 ng/ml IL-4 (BioLegend) for 24 h. Pharmacologic inhibition of p38 MAPK (SB203580; Sigma Aldrich) was achieved by pretreating BMDMs with 10 µM SB203580 for 1 h before addition of LPS or IL-4.

### Quantitative real-time PCR

Total RNA was extracted with TRIzol reagent (Invitrogen). cDNA was synthesized from 1 μg total RNA using an iScript cDNA synthesis kit (Bio-Rad). Quantitative real-time PCR (qPCR) was performed on a StepOnePlus Real-Time PCR system (ThermoFisher Scientific) using the PowerUp SYBR Green Master Mix (Thermo Fisher Scientific). Expression of target genes was normalized to *Hprt* mRNA and analyzed using the comparative C_T_ (ΔΔC_T_) method. The primer sequences are listed in Supplementary Table S1.

### Western blotting

Cells were lysed in NP-40 buffer containing 2% SDS and supplemented with protease and phosphatase inhibitor cocktail (Roche). Protein concentration was determined by BCA protein assay (ThermoFisher Scientific). Equal amount of protein was loaded and separated by SDS–PAGE followed by electrotransfer onto PVDF membrane (Millipore). The membranes were then incubated with primary antibodies at 4°C overnight, followed by incubation with horseradish peroxidase-conjugated secondary antibodies (Cell Signaling Technology). The proteins were visualized by Clarity Western ECL Substrate (Bio-Rad). The antibody information is listed in Supplementary Table S2.

### Flow cytometry

Freshly isolated tumor tissues were minced and digested with buffer consisting of 1% bovine serum albumin (BSA) and 1.5 mg/ml collagenase I (Sigma Aldrich) in PBS for 30 min at 37°C. Single-cell suspensions were filtered through 70-μm cell strainer, centrifuged at 500× *g* for 10 min at 4°C and resuspended in 10-ml FACS buffer that consists of 1% BSA (Sigma Aldrich) and 0.05% sodium azide (Sigma Aldrich). Approximately 1 × 10^6^ cells were stained with the fluorochrome-conjugated antibodies listed in Supplementary Table S2. To study BMDM polarization, bone marrow-derived cells were cultured in BMDM medium for 7 days and surface markers were stained with CD11b-PerCP-Cy5.5 and F4/80-APC. Data were acquired by an Attune NxT flow cytometer (Invitrogen).

### Histology and IHC

Tumor was dissected and fixed in 10% neutral buffered formalin (VWR) before paraffin embedding and sectioning. Then, 5-μm sections were deparaffinized, followed by antigen retrieval by boiling in a citrate-based antigen unmasking solution (Vector Laboratories) or Tris-EDTA buffer (Abcam) for 30 min. Tissue sections were permeabilized with 0.3% Triton X-100 (Sigma Aldrich) in PBS for 30 min and blocked with 2% horse serum (Gibco) for 30 min before incubating with primary antibodies overnight at 4°C. The antibody information is listed in Supplementary Table S2. After washing with PBS–0.1% Tween 20, the tissue sections were incubated with fluorescent dye-conjugated secondary antibodies (Invitrogen) at room temperature for 1 h. DAPI at 0.8 μg/ml was applied for 5 min, sections washed and coverslipped using fluorescence mounting medium (Dako). H&E staining was performed as described previously (Fischer et al., 2008). Images were acquired by an EVOS FL Auto Imaging System (ThermoFisher Scientific).

### Statistical analysis

All statistical analyses were performed using GraphPad Prism software. Data are expressed as mean ± standard error (SEM). Two-tailed unpaired Student’s *t*-test was used to determine statistical differences between two groups. Statistical differences between multiple groups were calculated using ANOVA followed by Tukey’s multiple comparison test. *P*-values <0.05 were considered significant, where the degree of statistical significance is defined as follows: *P < 0.05, **P < 0.01, and ***P < 0.001.

## Supplementary material

Supplementary material is available at *Journal of Molecular Cell Biology* online.

## Supplementary Material

mjaa038_Supplementary_material
