# Supplementary material for: MDMX phosphorylation-dependent p53 downregulation contributes to an immunosuppressive tumor microenvironment
Source: J Mol Cell Biol. 2020 Jul 24;12(9):713–22. doi: 10.1093/jmcb/mjaa038 (PMC7749742; doi:10.1093/jmcb/mjaa038)

MDMX phosphorylation-dependent p53 downregulation contributes to an immunosuppressive tumor microenvironment

Bing Wang[1, 2, 3, †], Chuan-Bian Lim[1, †], Jiawei Yan[1, 4], Lizhen Li[1], Jufang Wang[2], John B. Little[1], and Zhi-Min Yuan[1,*]

[1] John B. Little Center for Radiation Sciences, Harvard T.H. Chan School of Public Health, Boston, USA

[2] Present address: Key Laboratory of Space Radiobiology of Gansu Province & Key Laboratory of Heavy Ion Radiation Biology and Medicine of Chinese Academy of Sciences, Institute of Modern Physics, Chinese Academy of Sciences, Lanzhou, China

[3] Present address: University of Chinese Academy of Sciences, Beijing, China

[4] Present address: School of Life Sciences and Technology, ShanghaiTech University, Shanghai, China

[†] These authors contributed equally to this work.

* Correspondence to: Zhi-Min Yuan, E-mail: zyuan@hsph.harvard.edu

Tables

Supplementary Table 1 List of primers for qPCR, genotyping, and sequencing.

Primers	Sequence (5'-3')	qPCR	Genotyping	Sequencing
mHprt-F	GATCAGTCAACGGGGGACATAAAAG	+		
mHprt-R	CTGGCCTGTATCCAACACTTCGAG	+		
mNos2-F	TCTTGGAGCGAGTTGTGGATTG	+		
mNos2-R	GGTCGTAATGTCCAGGAAGTAGGTG	+		
mFpr2-F	GGTTGTCTCCATCACTTTCTTCCTTG	+		
mFpr2-R	GTGAACTAATTTACACAGGAACCAGCC	+		
mArg1-F	AAGAATGGAAGAGTCAGTGTGGTGC	+		
mArg1-R	GCTGGTTGTCAGGGGAGTGTTG	+		
mRetnla-F	AACTATCCCTCCACTGTAACGAAGACTC	+		
mRetnla-R	TGGTCCAGTCAACGAGTAAGCACAG	+		
Mdmx-S314A-F	CTATGAAATTTGTTCAGGTCTCAGGTTGGAC		+	
Mdmx-S314A-R	CTCCTACAATCGGGAACATCAATTCCTTC		+	
Mdmx-S314-gDNA-F	AGCTACCATGTGGTTGCTGGGAATTG			+
Mdmx-S314-gDNA-R	TAAGCTACACGGCTTCAAGACATTC			+
Mdmx-S314A-Seq	CGCTACCCAGTGGTTAAGAAC			+

Supplementary Table 2 List of antibodies for western blotting (WB), FACS, and immunofluorescence.

Antibody	Company	Catalog #	WB	FACS	Immunofluorescence
Actin (C4)	Santa Cruz	sc-47778	1:2000		
p53 (IC12)	CST	2524	1:1000		
p21 (12D1)	CST	2947	1:1000		
PUMA	Abcam	ab-9643	1:1000		
MDM2 (SMP14)	Santa Cruz	sc-965	1:1000		
CD45.1 (A20) PE	eBioscience	12-0453-82		1:200	
CD11b (M1/70) PerCP-Cy5.5	eBioscience	12-0112-82		1:200	
Ly-6G/Ly-6C (RB6-8C5) PE-Cy7	BioLegend	108416		1:200	
F4/80 (BM8) Alexa Fluo-488	BioLegend	123120		1:200	
F4/80 (T45-2342) APC	BD	566787		1:200	
CD45 (30F11)	Novus	NB100-77417			1:50
CD8 (32-M4)	Santa Cruz	sc-1177			1:50
MPO	R&D	AF-3667			10 μg/ml
iNOS	Novus	NB300-605			1:50
Arg1 (E-2)	Santa Cruz	sc-271430			1:50
p53 (CM5)	Leica	P53-CM5P-L			1:200
p21 (F-5)	Santa Cruz	sc-6246			1:200
ki67 (SP6)	Abcam	ab-16667			1:50

Supplementary Table 3 Analysis of mice from *Mdmx*$^{WT/S314A}$ × *Mdmx*$^{WT/S314A}$ cross.

	Expected frequency, % (*n*)	Observed frequency, % (*n*)	*P*-value
WT	25 (25/100)	27 (27/100)	
WT/S314A	50 (50/100)	52 (52/100)	0.6440
S314A/S314A	25 (25/100)	21 (21/100)	

Figure legends

Supplementary Figure S1 Mouse information. (**A**) Schematic illustration of the MDMX protein of mouse. Black arrow indicates the Ser314 phosphorylation site that was replaced by a nonphosphorylatable alanine by CRISPR-Cas9. S, serine; A, alanine. (**B**) Electropherogram obtained by sequencing the PCR products from mouse DNA of different genotypes. Black arrows indicate the serine codon TCT at position 314 that was mutated to an alanine codon GCA in the *Mdmx*$^{WT/S314A}$ and *Mdmx*$^{S314A/S314A}$ mice. WT, wild-type. (**C**) A silent mutation (AGC to TCG) was introduced as part of the genome editing strategy resulting in the generation of a novel TaqI restriction site that facilitated genotyping of animals. (**D**) Genomic PCR for genotyping. The PCR products were digested with TaqI to distinguish wild-type and S314A (TaqI) alleles. (**E**) Body weights of wild-type, *Mdmx*$^{WT/S314A}$, and *Mdmx*$^{S314A/S314A}$ mice. Body weights were taken weekly starting at 4 weeks of age until the animals were 20 weeks of age (n=5–7/genotype).

Supplementary Figure S2 EO771 cells were treated with 4 Gy X-ray for 3 h and harvested for immunoblotting with indicated antibodies.

Figure S1

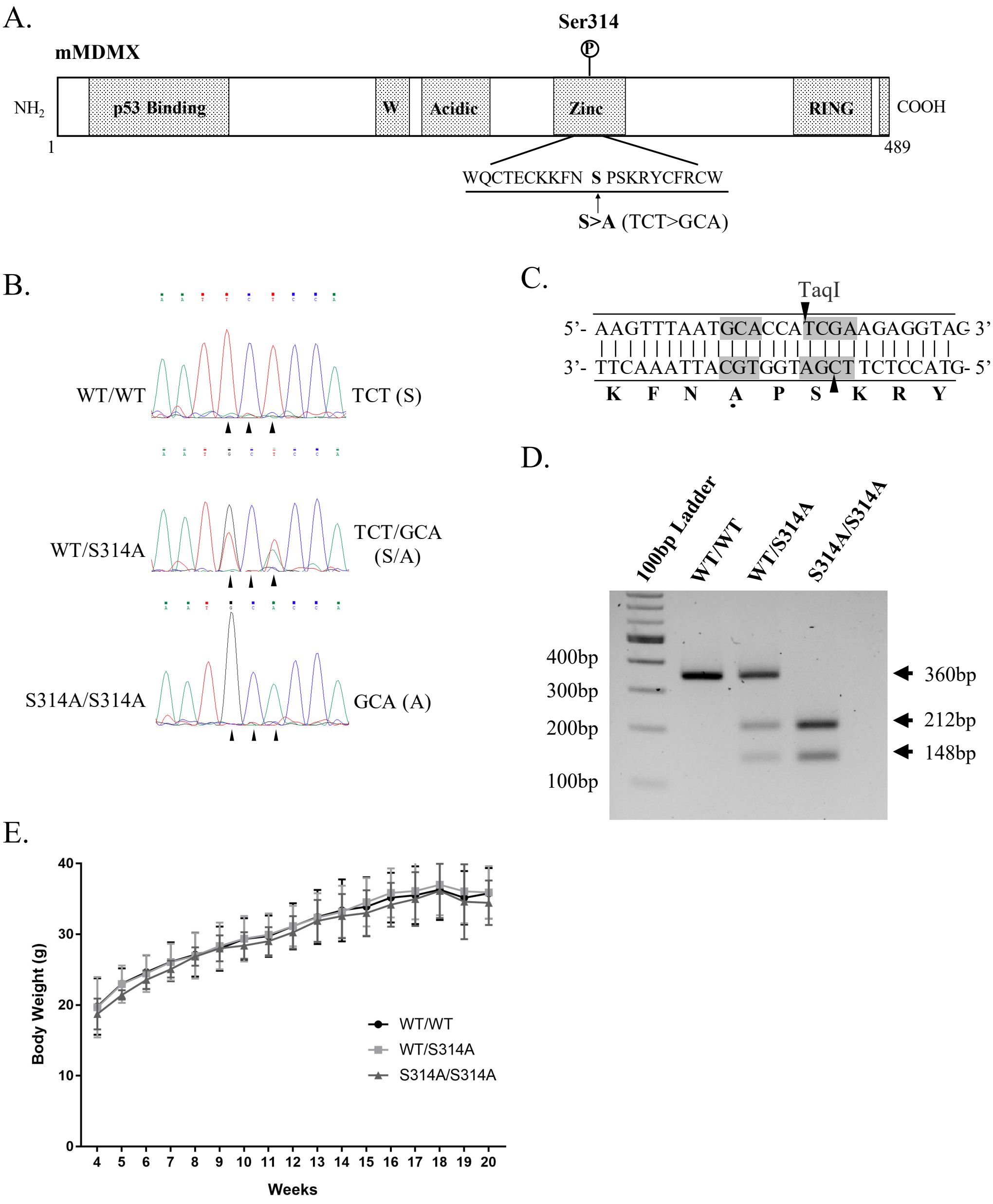
A.
Ser314
P
mMDMX
NH2
p53 Binding
W
Acidic
Zinc
RING
COOH
1
489
WQCTECKKFN S PSKRYCFRCW
S>A (TCT>GCA)
B.
WT/WT
TCT (S)
WT/S314A
TCT/GCA
(S/A)
S314A/S314A
GCA (A)
C.
TaqI
5'- AAGTTTAATGCACCATCGAAGAGGTAG 3'
3'- TTCAAATTACGTGGTAGCT TCTCCATG- 5'
K F N A P S K R Y
D.
100bp Ladder
WT/WT
WT/S314A
S314A/S314A
400bp
300bp
200bp
100bp
360bp
212bp
148bp
E.
Body Weight (g)
Weeks
WT/WT
WT/S314A
S314A/S314A
0
10
20
30
40
4
5
6
7
8
9
10
11
12
13
14
15
16
17
18
19
20

Figure S2

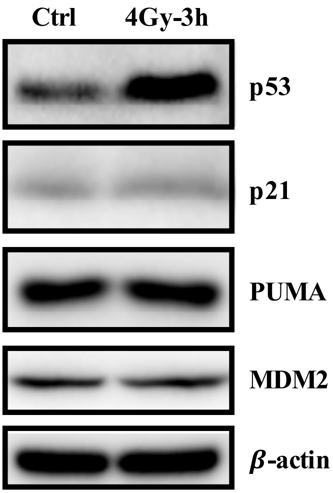

Supplement: mjaa038_Supplementary_material [file mjaa038_supplementary_material.pdf]
